# Carpenter Syndrome: Extended *RAB23* Mutation Spectrum and Analysis of Nonsense-mediated mRNA Decay

**DOI:** 10.1002/humu.21457

**Published:** 2011-02-08

**Authors:** Dagan Jenkins, Gareth Baynam, Luc De Catte, Nursel Elcioglu, Michael T Gabbett, Louanne Hudgins, Jane A Hurst, Fernanda Sarquis Jehee, Christine Oley, Andrew O M Wilkie

**Affiliations:** 1 Weatherall Institute of Molecular Medicine, University of Oxford, John Radcliffe HospitalOxford, United Kingdom; 2 Genetic Services of Western Australia, Princess Margaret Hospital for Children and King Edward Memorial Hospital for Women, School of Paediatrics and Child Health, University of Western AustraliaPerth, Australia; 3 Department of Obstetrics and Gynecology, University Hospital GasthuisbergLeuven, Belgium; 4 Department of Pediatric Genetics, Marmara University Medical FacultyIstanbul, Turkey; 5 Royal Brisbane & Women's Hospital, The University of QueenslandBrisbane, Australia; 6 The University of QueenslandBrisbane, Australia; 7 Division of Medical Genetics, Department of Pediatrics, Stanford UniversityStanford CA, USA; 8 Department of Clinical Genetics, Oxford Radcliffe Hospitals NHS TrustOxford, United Kingdom; 9 Centro de Estudos do Genoma Humano, Departamento de Genética e Biologia Evolutiva, Universidade de São PauloSão Paulo, Brazil; 10 West Midlands Regional Genetics Service, Birmingham Women's Healthcare NHS TrustBirmingham, United Kingdom

**Keywords:** *RAB23*, acrocephalopolysyndactyly syndrome, nonsense mediated mRNA decay, switch domain

## Abstract

Carpenter syndrome, a rare autosomal recessive disorder characterized by a combination of craniosynostosis, polysyndactyly, obesity, and other congenital malformations, is caused by mutations in *RAB23*, encoding a member of the Rab-family of small GTPases. In 15 out of 16 families previously reported, the disease was caused by homozygosity for truncating mutations, and currently only a single missense mutation has been identified in a compound heterozygote. Here, we describe a further 8 independent families comprising 10 affected individuals with Carpenter syndrome, who were positive for mutations in *RAB23*. We report the first homozygous missense mutation and in-frame deletion, highlighting key residues for RAB23 function, as well as the first splice-site mutation. Multi-suture craniosynostosis and polysyndactyly have been present in all patients described to date, and abnormal external genitalia have been universal in boys. High birth weight was not evident in the current group of patients, but further evidence for laterality defects is reported. No genotype-phenotype correlations are apparent. We provide experimental evidence that transcripts encoding truncating mutations are subject to nonsense-mediated decay, and that this plays an important role in the pathogenesis of many *RAB23* mutations. These observations refine the phenotypic spectrum of Carpenter syndrome and offer new insights into molecular pathogenesis. © 2011 Wiley-Liss, Inc.

## INTRODUCTION

Carpenter syndrome (MIM# 201000), a classical autosomal recessive multiple congenital malformation disorder first described in 1901 [Carpenter, 1901], is characterized by craniosynostosis, polysyndactyly, obesity and other malformations. We previously reported that Carpenter syndrome is caused by mutations in *RAB23* (MIM# 606144), encoding a member of the Rab-family of small GTPases involved in vesicle trafficking [Jenkins et al., 2007]. Since then, there has been a single confirmatory study describing a novel homozygous frameshift mutation in one family [Alessandri et al., 2010]. Because of a significant founder effect, with affected individuals in 10 out of 16 mutation-positive families being homozygous for a single mutation (c.434T>A encoding p.L145X, present on an apparently shared haplotype), the spectrum of described mutations in *RAB23* is limited. The collated *RAB23* mutation data document homozygosity for five different truncating mutations (three associated with frameshifts and two encoding nonsense codons) in all but one of the 16 families reported to date; the final affected individual being a compound heterozygote for p.L145X and a predicted missense mutation, p.C85R [Jenkins et al., 2007; Alessandri et al., 2010]. Currently it is unclear whether the lack of homozygous missense mutations is a chance observation or possibly reflects a distinct functional effect of such mutations, leading to a different phenotype.

**Figure 1:**
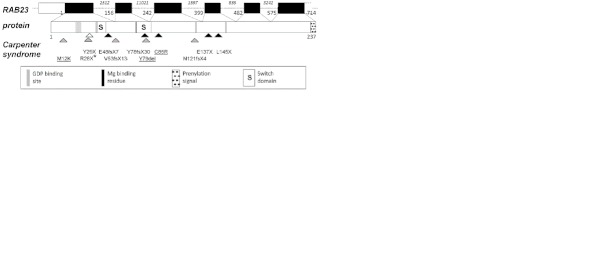
Mutation spectrum in *RAB23*. At top, the exon/intron organization of *RAB23*, with the coding part of the cDNA in black and the untranslated regions (UTRs) in white (only exons 2-7 are shown; alternatively spliced 5′ noncoding exons are omitted). Upright numbering refers to the first nucleotide of each exon, starting from the initiation codon, and italic numbering indicates the length of introns. Below, the functional domains in the protein are depicted according to the key. The location of all human mutations described to date that cause Carpenter syndrome are indicated by arrowheads. Black and white arrowheads refer to mutations reported by Jenkins et al. [2007] and Alessandri et al. [2010], respectively. Grey arrowheads refer to novel alleles reported in the current study, and mutations affecting single amino acids are underlined. The asterisk refers to the fact that the p.R28X mutation was co-inherited with the p.L145X mutation, giving a p.[R28X;L145X] allele. Nucleotide numbering reflects cDNA numbering with +1 corresponding to the A of the ATG translation initiation codon in the reference sequence, according to journal guidelines (http://www.hgvs.org/mutnomen). The initiation codon is codon 1.

All Rab proteins have the same arrangement of functional domains (Figure 1). These include several regions that come together in the three dimensional structure of the protein to form a GTP/GDP binding pocket, and two so-called ‘switch’ domains that interact with Rab-effector proteins and undergo a conformational change according the presence of either GDP or GTP. Rab proteins also contain a C-terminal prenylation motif, consisting of the last four amino acids; after translation, lipid modification occurs at this motif following geranylgeranylation, which is essential for targeting of Rabs to specific membranes, and thus for their subsequent function [Pfeffer & Aivazian, 2004]. Whereas most Rab proteins have a dicysteine prenylation motif, that of Rab23 has only a single cysteine residue, more characteristic of the Rho and Ras GTPase families. Because of this, and unlike other Rabs with dicysteine motifs, Rab23 is not trafficked through the secretory pathway [Leung et al., 2007]. Instead, trafficking of Rab23 to the plasma membrane may involve an alternative mechanism involving phospholipids [Heo et al., 2006]. Given the current absence of homozygous point mutations in *RAB23*, it is not yet clear how disruption of these different functional domains contributes to pathogenesis.

As well as disruption of protein function, the other mechanism to be considered in Carpenter syndrome is nonsense-mediated mRNA decay (NMD). NMD is a surveillance mechanism that recognizes and degrades transcripts carrying premature termination codons (PTCs) arising from erroneous transcription or splicing, or that are encoded by splice-site or nonsense/frameshift mutations [Khajavi et al., 2006]. NMD has been shown to modulate human disease phenotypes. For example, the clearance of mutant transcripts by NMD may reduce disease severity by eliminating transcripts that encode proteins with dominant-negative effects. Disease phenotypes associated with NMD may also depend on the position of a PTC within the gene, because PTCs are recognized by the presence of exon junction complexes at downstream exon-exon junctions. Therefore, PTCs may escape NMD if they are located in the final exon or 3′ end of the penultimate exon. For example, nonsense mutations in the final exon of either *HBB* or *SOX10* escape NMD and have dominant-negative effects, thereby giving rise to more severe phenotypes than nonsense mutations located in upstream exons that are subject to NMD [Hall & Thein, 1994; Inoue et al., 2004]. The stability of *RAB23* transcripts carrying PTCs has not previously been investigated.

## MATERIALS AND METHODS

### Identification of RAB23 mutations

This study was approved by the Oxfordshire Research Ethics Committee B (C02.143) and informed consent was obtained from the parents of affected children. Genomic DNA was extracted from peripheral blood by proteinase K digestion and phenol-chloroform extraction. All coding exons (exons 2-7) of *RAB23*, and their surrounding intronic regions, were sequenced in each patient in both forward and reverse directions as described previously [Jenkins et al., 2007]. Mutation nomenclature is based on http://www.hgvs.org/mutnomen/recs-prot, and nucleotide numbering of *RAB23* cDNA is based on GenBank sequence NM_183227.1, starting from the first base of the initiation codon.

### Analysis of abnormal splicing

Total RNA was extracted from peripheral blood (obtained in PAXgene tubes) using the PAXgene Blood RNA Kit (QIAGEN, Crawley, UK), from which cDNA was prepared by reverse transcription using the RETROscript Kit (Ambion/Applied Biosystems, Warrington, UK). Reverse transcriptase-PCR (RT-PCR) was performed using primers (Forward – 5′-TCGCCATAAAGATGGTGGTTGTAGGGAATG-3′ and Reverse – 5′-GCACAAGTACAGTTGGTATATCTCCCACTTC-3′), located in exons 2 and 4, respectively.

### Quantification of NMD by pyrosequencing

To analyze the c.434T>A (p.L145X) mutation, cDNA was prepared from peripheral blood as described above, and a 304 bp RT-PCR product spanning *RAB23* exons 3 to 6 was generated using the following primers: Forward – 5′-GCTTGTGTGCTCGTGTTCTC-3′ and Reverse – 5′-GCGTTAGTTCTGGATCCTCAG-3′. Single-stranded DNA was obtained from 10 μl of each of three independent PCR products by immobilization on streptavidin-coated sepharose beads (Streptavidin Sepharose high performance, GE Healthcare, Chalfont St. Giles, UK), and denatured using NaOH. Pyrosequencing was performed on a PyroMark Q96 MD (QIAGEN, Crawley, UK) in the reverse direction using the primer 5′-CTGATGTTCTGTAGAATCTT-3′. After dispensation of enzyme (E) and substrate (S), the nucleotides were dispensed in the order A-T-C-A-T-C-G-C-A-T-C-A-C-T-G-C. Dispensations were designed to generate several peaks unique to either mutant or wild-type alleles, as well as blank peaks that were negative for both mutant and wild-type alleles, so as to measure background peak heights. All pyrograms passed the following quality control criteria: (1) mutant-specific peaks were not generated in wild-type cDNA samples (mutant/wild-type (M/WT) ratio <0.05); (2) deliberately blank dispensations did not produce peaks. Following subtraction of blank peaks, M/WT ratios were calculated for peaks generated by the same nucleotide dispensed at similar positions.

## RESULTS

**Figure 2:**
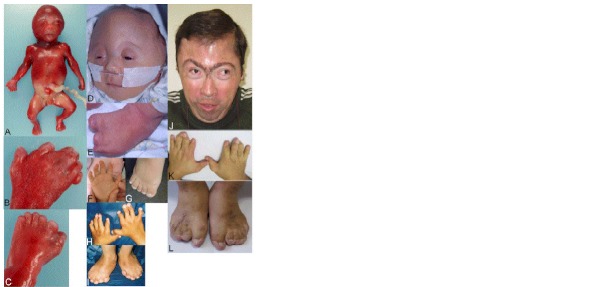
Clinical appearance of *RAB23* mutation-positive Carpenter syndrome at different ages. (**A-C**) Subject 4388, pregnancy terminated at 19.5 weeks' gestation. (**D-I**) Childhood pictures of subjects 4206/7 aged 3 days (**D,E**), 4203 aged 11 mo (**F**) and 3 yr (**G**) and 4119 aged 7 yr (**H,I**). (**J,K,L**) Subject 4121 aged 29 years. Note dysmorphic craniofacial appearance (**A,D,J**) and variable combinations of polydactyly, syndactyly and brachydactyly (**B,C,H,I,K,L**). However polysyndactyly was absent in subject 4203 homozygous for the p.M12K missense mutation (**F,G**).

As part of our ongoing screening of patients with Carpenter syndrome, we identified a further 10 subjects, from 8 independent families, with biallelic mutations in *RAB23*. A summary of the mutations is presented in Table 1 and Figure 1; associated clinical features are detailed in Supp. Table S1 (available as a separate Supporting Information file) and representative clinical photographs are provided in Figure 2. All patients were born to phenotypically normal parents and, when samples were available for analysis, heterozygosity for the identified mutations was confirmed in these parents.

**Table 1:** *RAB23* mutations identified in patients with Carpenter syndrome in the current study

					Mutation at Allele			

					Maternal		Paternal	

Subject	Sex	Parental Consanguinity	Country of Origin	Sample(s) Analyzed	DNA	Protein	DNA	Protein
4203	F	-	Mexico	P, Mo and Fa	c.35T>A	p.M12K	c.35T>A	p.M12K

4388 (Family 2)	M	-	north European (Australia)	P, Mo and Fa	c.[82C>T; 434T>A]	p.[R28X;L145X]	c.434T>A	p.L145X

4389 (Family 2)	M	-	north European (Australia)	P, Mo and Fa	c.[82C>T; 434T>A]	p.[R28X;L145X]	c.434T>A	p.L145X

4154	M	-	north European (Belgium)	P, Mo and Fa	c.434T>A	p.L145X	C.156-3T>G	p.V53fsX13

4119 (Family 1)	M	1st cousins	Turkey	P, Mo and Fa	c.234_236delCTA	p.Y79del	Same	-

4120 (Family 1)	M	1st cousins	Turkey	P, Mo and Fa	c.234_236delCTA	p.Y79del	Same	-

4121	M	1st cousins	Brazil	P and Mo	c.362 363insG	p.N121fsX4	Same	-

554	F	-	north European (UK)	P	c.434T>A	p.L145X	?	?

4080	M	-	north European (UK)	P	c.434T>A	p.L145X	?	?

4206/7	M	-	north European (Australia)	P, Mo and Fa	c.434T>A	p.L145X	c.434T>A	p.L145X

Abbreviations: F, female; Fa, father; M, male; Mo, mother; P, patient. For cases in which the two parental alleles are unlikely to be independent (owing to documented consanguinity), the paternal allele is denoted “Same”. Mutations for which only the patient DNA was available are listed in the Maternal columns, with “?” in the Paternal columns.

We identified 6 different *RAB23* mutant alleles in this series, all of which are novel except for the common c.434T>A (p.L145X) mutation. p.L145X comprised at least one mutant allele in all 5 families of white north European origin, consistent with the founder effect previously identified in this population. In 3 of these families (subjects 554, 4080 and 4206/7), affected individuals were apparently homozygous. In the other two north European families affected individuals were compound heterozygotes for L145X and a different allele; in one (siblings 4388 and 4389), two different nonsense mutations (p.[R28X;L145X]) were co-inherited from the unaffected mother, in the other (subject 4154) a novel mutation, c.156-3T>G, which causes disrupted splicing, was present (see below).

In each of the three families originating outside of northern Europe, we identified a different, novel homozygous mutation. A patient from Brazil (subject 4121) had a frameshift mutation, p.N121fsX4, similar to others that we described previously. The three other patients had mutations affecting single amino acid residues (subject 4203 from Mexico, c.35T>A encoding p.M12K; siblings 4119 and 4120 from Turkey, c.234_236del encoding p.Y79del). Both mutations are expected to affect protein function on the basis of evolutionary conservation, crystal structure and domain organization of RAB23 (Figure 1 and Supp. Figure S1; Eathiraj et al., 2005; see *Discussion* below).

**Figure 3:**
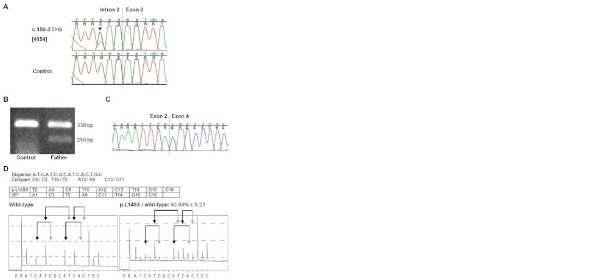
Analysis of transcripts encoded by a splice-site or nonsense mutation. **(A)** Sequencing of genomic DNA from subject 4154 showing a heterozygous T>G substitution (arrowhead) 3 bp upstream of exon 3. **(B)** RT-PCR with primers in exons 2 and 4 using cDNA extracted from fresh blood of the father of subject 4154. As well as a wild-type fragment at 336 bp, a second fragment is also observed at 250 bp, which is absent in control cDNA. Note that the smaller fragment is much weaker than the wild-type product. **(C)** Sequencing of the 250 bp fragment, demonstrating skipping of exon 3 in the cDNA product. **(D)** Pyrosequencing assay used to measure the relative levels of c.434T>A (p.L145X) mutant and wild-type transcripts. The dispensation order and peaks for comparison are indicated, below which is a table listing the sequence of nucleotides incorporated into mutant and wild-type transcripts, and the position at which each peak is produced (for example T2 indicates that a T is incorporated at the second dispensation). Representative pyrograms are shown using cDNA originating from peripheral blood of a wild-type individual (left) and a parent heterozygous for the mutation (right). Pairs of arrows link peaks (black: wild-type; grey, mutant) used for comparative quantification.

The c.156-3T>G mutation present in subject 4154 is located just upstream of exon 3 (Figure 3 A). Mutations of the -3 position are a relatively unusual cause of abnormal splicing, but G at −3 is strongly disfavored at 3′ splice sites and precedents exist for this change being pathogenic [Wong et al., 1989]. To determine whether this mutation affects splicing, RT-PCR was performed using primers in exons 2 and 4. Amplification of control *RAB23* cDNA generated a single 336 bp product, as expected for a full-length transcript that includes exon 3. By contrast, analysis of cDNA extracted from peripheral blood obtained from the father (from whom the mutation had been inherited), showed an additional smaller product of ∼250 bp, consistent with skipping of exon 3 (Figure 3B). Gel purification and direct sequencing confirmed that this smaller fragment comprised exon 4 spliced directly to exon 2, producing an out-of-frame transcript which introduces a PTC (designated r.156_241del, predicting the frameshift mutation p.V53fsX13) (Figure 3C). Notably, the intensity of the 250 bp fragment was significantly less than the 360 bp wild-type fragment, despite its smaller size; this suggests that the mutant transcript is unstable.

To investigate the wider significance of NMD for expression of *RAB23* transcripts, we used pyrosequencing to quantify the relative levels of wild-type and mutant transcripts in the mother of a patient who we previously reported with Carpenter syndrome (subject 3734; [Jenkins et al., 2007]), who was heterozygous for the c.434T>A (p.L145X) allele (Figure 3D). There was a clear reduction in the levels of mutant transcripts compared to wild-type; we determined that transcripts encoding p.L145X were present at 43.8% ± 8.2% (standard error of the mean) of the level of wild-type transcripts, suggesting that the p.L145X allele also confers transcript instability.

## DISCUSSION

In this study, we have identified a further 8 families with mutations in the *RAB23* gene, nearly doubling the number of different mutant alleles known. These additional patients have enabled us to establish a clearer picture of the phenotypic spectrum in Carpenter syndrome. We provide the first direct evidence that truncating mutations in *RAB23* are unstable, suggesting a role for NMD in pathogenesis, and describe the first homozygous mutations in *RAB23* that encode single amino acid changes. These mutations are associated with a similar phenotypic spectrum to nonsense mutations, thereby identifying key residues that are essential for protein function.

The cases presented here, together with those previously published by Jenkins et al. [2007] and Alessandri et al. [2010] provide an expanded series in which to examine the phenotypic spectrum of Carpenter syndrome. The only universal features are craniosynostosis and soft-tissue syndactly, usually accompanied by insertional/preaxial polydactyly of the feet and often postaxial polydactyly of the hands; cryptorchidism/hypoplastic external genitalia were observed in almost all male cases (Supp. Table S1). Where CT scanning of the skull was performed, fusion of the sagittal suture was observed in all patients, and bicoronal synostosis was almost always present. Whereas all patients in the study by Jenkins et al. [2007] had high birth weight, the birth weights in the current group of patients were in the normal range, although they did go on to exhibit postnatal obesity. Polysplenia/accessory spleens, a characteristic laterality defect, was previously reported by Alessandri et al. [2010] in a single patient. One patient in the present study (subject 4080) exhibited this phenotype, confirming this as a specific association with mutation of *RAB23*, and suggesting a role for RAB23 in the regulation of left-right patterning.

Greig syndrome, which is caused by heterozygous loss-of-function mutations in *GLI3* (MIM# 165240), is also characterized by polysyndactyly, as well as other features seen in Carpenter syndrome, including agenesis of the corpus callosum and cryptorchidism in boys [Johnston et al., 2005]. Recently, synostosis of the metopic or sagittal sutures has been reported as a rare association with Greig syndrome [Johnston et al., 2010; Kini et al., 2010; McDonald-McGinn et al., 2010], further confusing accurate diagnosis. Indeed, three patients referred to us for *RAB23* mutation testing proved instead to be positive for 6.0-8.3 Mb deletions including *GLI3* [Hurst et al., 2011]. Currently, only midline synostosis has been reported in Greig syndrome, so the presence of bicoronal synostosis in almost all patients with Carpenter syndrome, together with other frequent features such as obesity and umbilical hernia, help to distinguish between the two disorders. However, patients with either diagnosis should be considered for both *GLI3* and *RAB23* testing.

The identification of a homozygous missense mutation (p.M12K) and a homozygous amino acid deletion (p.Y79del) in RAB23, which were both associated with fairly typical features of Carpenter syndrome, highlights two residues important for normal biochemical function. Like other Rab proteins, the core structure of RAB23 comprises 6 buried β-strands [Eathiraj et al., 2005]. Both the p.M12K mutation (Supp. Figure S1A) and the previously reported p.C85R mutation [Jenkins et al., 2007], represent non-conservative substitutions from sulfur-containing amino acids located within this core, to charged residues with more bulky side chains. These mutations are therefore likely to disrupt protein folding. The p.Y79del is located within one of the switch domains of RAB23. Both Rab3a and Rab5c contain a triad of hydrophobic residues, including a tyrosine residue in the switch domain, equivalent to Y78 in RAB23, that interact with effector proteins [Merithew et al., 2001]. The Y78 residue is adjacent to the deleted Y79, which normally points in the opposite direction and is conserved in both Rab3a and Rab5c (Supp. Figure S1B). Therefore, deletion of the Y79 residue is likely to disrupt interaction of RAB23 with effector proteins. Because patients homozygous for both p.M12K and p.Y79del exhibit the full Carpenter syndrome phenotype, it is likely that these mutations result in complete loss-of-function.

Notwithstanding these considerations, most patients with Carpenter syndrome reported to date are homozygous for truncating (nonsense or frameshift) mutations in RAB23. Interestingly, these truncating mutations seem to be distributed unevenly, being absent in the final third of the protein (Figure 1). If the C-terminal prenylation motif of RAB23 were essential for function, as in other Rabs, then truncating mutations located anywhere in the gene would be expected to generate null-alleles. Although further mutation screening will be required to confirm that the spectrum of truncating mutations is non-random, this may be explained by the fact that the prenylation motif of RAB23 is atypical (see *Introduction*). This suggests that alternative mechanisms, other than disruption of prenylation, may also be critical for pathogenesis; NMD represents one such mechanism.

We evaluated NMD by analyzing the relative levels of mutant and wild-type transcripts in individuals heterozygous for *RAB23* mutations, and found that transcripts encoding truncated forms of *RAB23* are unstable. This was demonstrated both for a splice site mutation (PTC located in the third of six coding exons) and for the most 3′ (and most common) mutation in *RAB23* (p.L145X; PTC in fourth coding exon), suggesting that all truncating mutations in Carpenter syndrome are likely to undergo NMD. Because PTCs in the last exon and 3′ part of the penultimate exon of *RAB23* encoding residues downstream of p.L145, are expected to escape NMD [Khajavi et al., 2006], the absence of mutations in this region may also reflect a critical role for NMD in pathogenesis. Although more N-terminally located truncating mutations might be expected to give rise to more severe phenotypes by disrupting more of the functional domains, no genotype-phenotype correlations are apparent with respect to the location of truncating mutations in *RAB23*. This evidence also points to an important role for NMD in pathogenesis.

## Supplementary material

**Supp. Figure S1.** Location of residues altered by homozygous mutations of single amino acids in *RAB23*. (**A**) Crystal structure of RAB23 (Protein Databank accession 1Z22) showing the location of the p.M12K mutation in the first β-strand (red arrow). Below, the p.M12 residue (highlighted in red) is conserved as an uncharged amino acid in *Homo sapiens* (Hs), *Mus musculus* (Mm), *Danio rerio* (Dr) and *Drosophila melanogaster* (Dm). (**B**) Same crystal structure of RAB23 rotated compared to **A** to show three residues (Y78; W63; F46) that form a hydrophobic triad that interacts with effector proteins (white arrows); p.Y79 is indicated by the red arrow. Below, the p.Y79 residue (highlighted in red) and surrounding amino acids are highly conserved in evolution.
