# Supplementary figures and images for: Carpenter Syndrome: Extended RAB23 Mutation Spectrum and Analysis of Nonsense-mediated mRNA Decay
Source: Hum Mutat. 2011 Feb 8;32(4):E2069–78. doi: 10.1002/humu.21457 (PMC3429868; doi:10.1002/humu.21457)

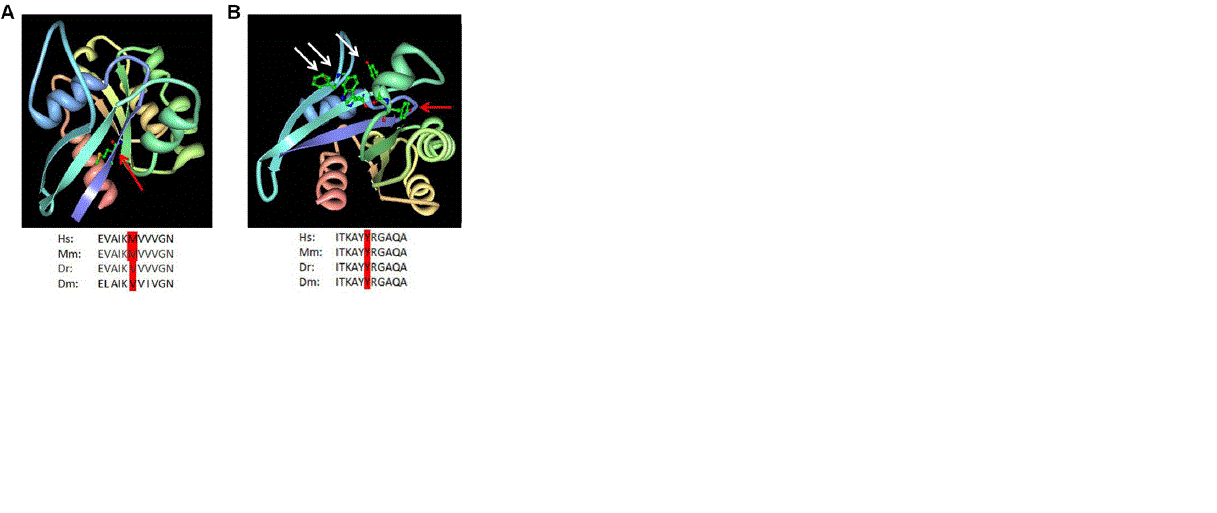

Supplement: Supplementary file 1 [file humu0032-E2069-SD1.gif]
